# Model-Based Planning Deficits in Compulsivity Are Linked to Faulty Neural Representations of Task Structure

**DOI:** 10.1523/JNEUROSCI.0031-21.2021

**Published:** 2021-07-28

**Authors:** Tricia X. F. Seow, Edith Benoit, Caoimhe Dempsey, Maeve Jennings, Aoibheann Maxwell, Redmond O'Connell, Claire M. Gillan

**Affiliations:** ^1^Department of Psychology, Trinity College Dublin, Dublin 2, Ireland; ^2^Trinity College Institute of Neuroscience, Trinity College Dublin, Dublin 2, Ireland; ^3^Global Brain Health Institute, Trinity College Dublin, Dublin 2, Ireland

**Keywords:** compulsivity, electroencephalography, goal-directed learning, model-based learning, reinforcement learning, transdiagnostic psychiatry

## Abstract

Compulsive individuals have deficits in model-based planning, but the mechanisms that drive this have not been established. We examined two candidates—that compulsivity is linked to (1) an impaired model of the task environment and/or (2) an inability to engage cognitive control when making choices. To test this, 192 participants performed a two-step reinforcement learning task with concurrent EEG recordings, and we related the neural and behavioral data to their scores on a self-reported transdiagnostic dimension of compulsivity. To examine subjects' internal model of the task, we used established behavioral and neural responses to unexpected events [reaction time (RT) slowing, P300 wave, and parietal-occipital alpha band power] measured when an unexpected transition occurred. To assess cognitive control, we probed theta power at the time of initial choice. As expected, model-based planning was linked to greater behavioral (RT) and neural (alpha power, but not P300) sensitivity to rare transitions. Critically, the sensitivities of both RT and alpha to task structure were weaker in those high in compulsivity. This RT-compulsivity effect was tested and replicated in an independent pre-existing dataset (*N* = 1413). We also found that mid-frontal theta power at the time of choice was reduced in highly compulsive individuals though its relation to model-based planning was less pronounced. These data suggest that model-based planning deficits in compulsive individuals may arise, at least in part, from having an impaired representation of the environment, specifically how actions lead to future states.

**SIGNIFICANCE STATEMENT** Compulsivity is linked to poorer performance on tasks that require model-based planning, but it is unclear what precise mechanisms underlie this deficit. Do compulsive individuals fail to engage cognitive control at the time of choice? Or do they have difficulty in building and maintaining an accurate representation of their environment, the foundation needed to behave in a goal-directed manner? With reaction time and EEG measures in 192 individuals who performed a two-step decision-making task, we found that compulsive individuals are less sensitive to surprising action–state transitions, where they slow down less and show less alpha band suppression following a rare transition. These findings implicate failures in maintaining an accurate model of the world in model-based planning deficits in compulsivity.

## Introduction

Compulsive behavior manifests as out-of-control and repetitive actions, often leading to functionally impairing outcomes (Robbins et al., 2012). This symptomology is characteristic of psychiatric disorders like obsessive-compulsive disorder (OCD) and addiction, and is thought to arise from an imbalance between the following two modes of action control (Gillan and Robbins, 2014): (1) goal-directed “model-based” (MB) planning relying on knowledge of how actions lead to specific outcomes and (2) rigid habits depending on reflexive stimulus–response associations that form slowly over time (Dickinson, 1985; Balleine and O'Doherty, 2010). The compulsivity literature has largely focused on testing whether a dysfunctional imbalance in the competitive interactions between these decision systems causes habitual behaviors to dominate (Lee et al., 2014; Gruner et al., 2016); but rather than being solely an arbitration failure, recent evidence suggest that compulsivity may be primarily associated with goal-directed control impairments. For example, OCD patients have performance deficits in the two-step reinforcement task (Voon et al., 2015) where model-based planning, a reinforcement-learning model of goal-directed action, is operationalized as the extent to which individuals make decisions using knowledge of how their actions relate to subsequent events (Daw et al., 2005, 2011). Recent work has shown that this dysfunction has a developmental course (Vaghi et al., 2020) and is best captured by a compulsivity dimension in both general population and patient samples (Gillan et al., 2016).

However, it remains unclear what underlies model-based planning problems in compulsivity—a multifaceted cognitive capacity, model-based planning depends on several functions including: (1) the construction/maintenance of an internal model (i.e., a representation of the environment, like the knowledge of relevant action–outcome and state–state relationships), which is a prerequisite for (2) implementation of this model in behavior through prospective planning. Model-based failures could theoretically stem from mechanistic issues underlying either component (and others that are not the focus of the present study). Though direct tests to resolve this have been lacking, patients show goal-directed deficits even when they have explicit knowledge of simple action–outcome contingencies (Gillan et al., 2014), suggesting that OCD patients may have issues solely with implementation. But, paradigms that feature more numerous and/or taxing contingency structures revealed problems in learning action–outcome associations in OCD and addiction (Gillan et al., 2011; Ersche et al., 2016), which correlated with goal-directed control failures in OCD (Gillan et al., 2011). Overall, the evidence remains equivocal because these devaluation-style tasks conflate goal-directed control deficits with increases in stimulus–response habit learning (Watson and de Wit, 2018) and were not designed to assess participants' ability to represent the task environment.

Recent data has suggested that goal-directed failures in compulsivity might arise from the latter. For example, compulsivity is linked to poorer learning of the consequences of actions (Sharp et al., 2020), and at the meta-level, highly compulsive individuals have abnormalities in how they view their own actions, exhibiting an overconfidence, which is relatively impervious to corrective evidence (Rouault et al., 2018; Seow and Gillan, 2020). Though studied in a different context, these findings suggest the possibility that individuals high in compulsivity have fundamental issues in acquiring and maintaining an accurate internal model of the world. To date, no study has examined neural representations of task structure in compulsive individuals as they perform a model-based planning task. The present study aimed to fill this gap—testing whether compulsivity is characterized by a disruption in constructing/maintaining an accurate representation of the task environment, or the use/implementation of this model in their choices. To do this, we analyzed reaction time (RT) and electroencephalography (EEG) data to define signatures of state transition knowledge (RT, P300, and posterior alpha) and of a well established cognitive control marker (mid-frontal theta) as 192 subjects performed a two-step reinforcement learning task (Daw et al., 2005, 2011). With single-trial regression analyses, we sought to characterize several candidate neural correlates of the representation and implementation of the mental model and to test whether they associated with individual differences in model-based planning and compulsivity.

## Materials and Methods

### 

#### 

##### Power estimation.

We determined a minimum sample size from a prior study that investigated the association of goal-directed control (on a different task) with Obsessive-Compulsive Inventory–Revised (OCI-R) scores from nonclinical participants who were also tested in person (*r* = −0.26, *p* < 0.05; Snorrason et al., 2016). The effect size indicated that *N* = 150 participants were required to achieve 90% power at 0.05 significance. Our final sample was larger than this to achieve the required power for another study that the same subjects participated in (Seow et al., 2020).

##### Participant exclusion criteria.

During recruitment, all participants were ensured to be ≥18–65 years of age, and had no personal/familial history of epilepsy, no personal history of neurologic illness/head trauma, or unexplained fainting. Participants' data were excluded from analysis if they failed any of the following on a rolling basis: (1) participants whose EEG data were incomplete (*N* = 5; i.e., recording was prematurely terminated before the completion of the task) or corrupted (*N* = 2); (2) participants whose EEG data contained excessive noise (i.e., >70% EEG epochs from the individual failing epoch exclusion criteria; see *EEG recording and preprocessing*; *N* = 4); (3) participants who responded with the same key in stage 1 >90% (*n* > 135 trials) of the time (*N* = 10); (4) participants whose probability of staying after common-rewarded trials was significantly worse than chance, which was measured as <5% probability of fitting a binomial distribution with 50% (chance) probability and the total number of common-rewarded trials experienced by each subject (*N* = 11); (5) participants who missed >20% of trials (*n* > 30 trials; *N* = 3); and (6) participants who incorrectly responded to the following “catch” question within the questionnaires: “If you are paying attention to these questions, please select 'A little' as your answer” (*N* = 7). Combining all exclusion criteria, 42 participants (17.95%) were excluded with *N* = 192 participants left for analysis [115 females (59.90%), between 18 and 65 years of age (mean age = 31.55 years, SD = 11.75 years)]. Excluded participants did not significantly differ in any of the three psychiatric dimension scores (see *Self-report psychiatric questionnaires, transdiagnostic dimensions and IQ*; all *p* values >0.06) from participants whose data were analyzed.

##### Procedure.

Before presenting to the laboratory for in-person EEG testing, participants completed a brief at-home assessment via the internet. They provided informed electronic consent and submitted basic demographic data (age and gender), listed any medication they were taking for a mental health issue, and completed a set of nine self-report psychiatric questionnaires (see *Self-report psychiatric questionnaires, transdiagnostic dimensions and IQ*). During the in-person EEG session, participants completed the following two tasks: the modified Eriksen flanker task (Eriksen and Eriksen, 1974) and the two-step reinforcement learning task (Daw et al., 2005, 2011). Data from the former task have been published previously (Seow et al., 2020), but note that we also reported the basic association with compulsivity and model-based planning in that article, which served to contextualize a null result. Once participants had completed both tasks, they completed a short IQ evaluation before debriefing. A subset of the participants (*N* = 110, 47%) completed a short psychiatric interview [Mini International Neuropsychiatric Interview, English Version 7.0.0 (M.I.N.I.); Sheehan et al., 1998] before the experimental tasks to establish their diagnostic status.

##### Disorder prevalence (M.I.N.I.).

After exclusion, 80 participants (41.67%) completed the M.I.N.I., which was introduced partway through the study to add additional clinical context above our self-report measures. Of these participants, 35 (43.75%) met the criteria for one or more disorder. Broken down by recruitment arm, all 7 subjects (100%) recruited from the clinical setting met the criteria, while 28 subjects (38.36%) from university channels met the criteria. This rate is close to those in published reports on the prevalence of mental health disorders in college student samples (Auerbach et al., 2018; Evans et al., 2018). Of the total sample, 33 subjects (17.19%) were currently receiving medication for a mental health issue. Broken down by recruitment arm, all individuals recruited from the clinic were receiving medication, while 26 (14.05%) of those recruited through normal channels were receiving medication.

##### Two-step reinforcement learning task.

**Figure 1.**
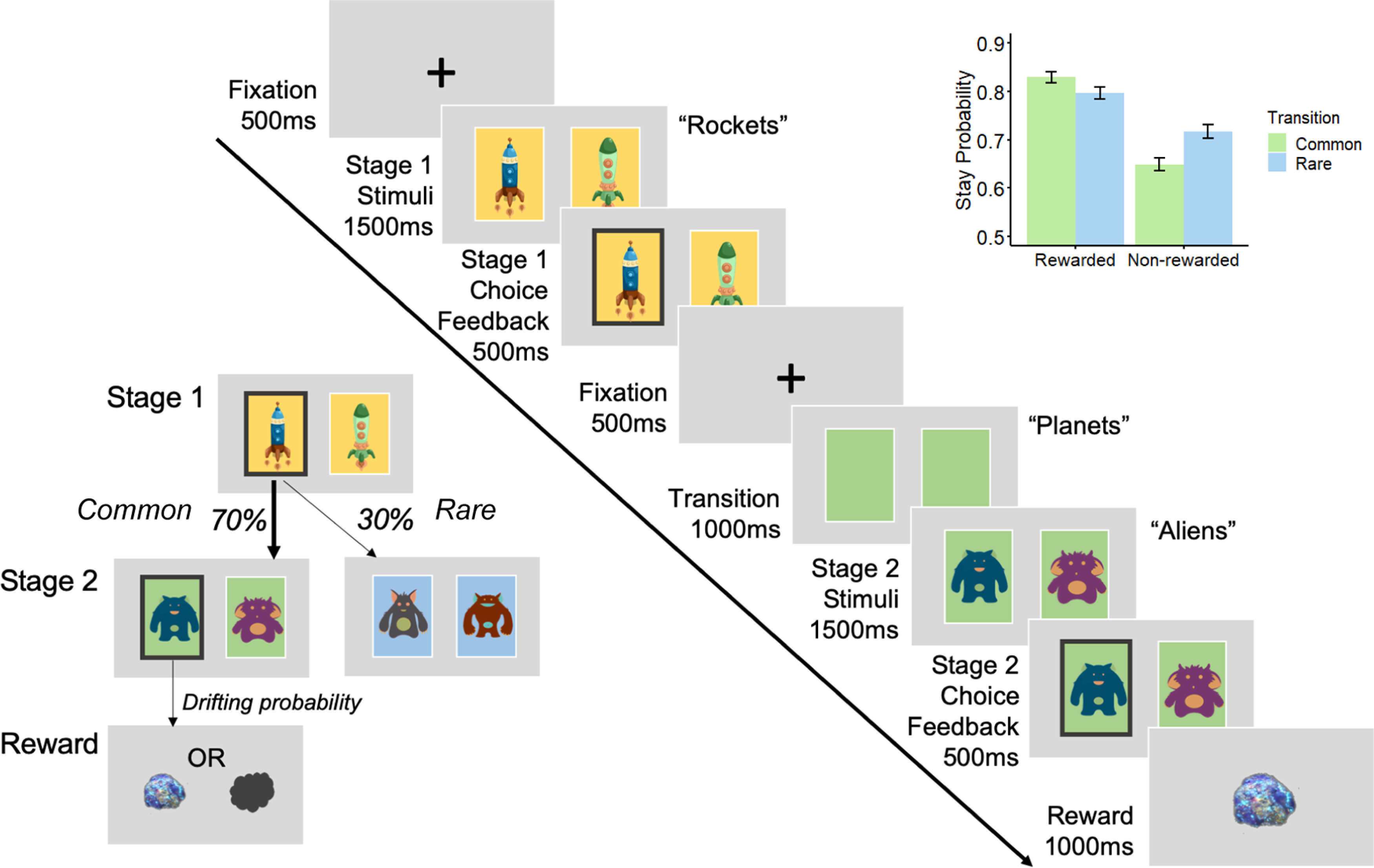
Two-step reinforcement learning task. The paradigm consists of two stages where participants take a rocket that has a common (70%) or rare (30%) 'transition probability' of taking them to one of two second-stage planets (states). Aliens on these planets each have a unique probability of reward [space treasure (reward) or space dust (nonreward)] that drifts slowly throughout the entire experiment. Participants have to take into consideration the task transition structure and their history of rewards to make choices that maximize reward. The sequence of events as presented for EEG is the same as that of Eppinger et al. (2017), except they included a manipulation of transition probabilities in their study (comparing 60%/40% to 80%/20%) and used a longer choice window (2000 ms). On the top right inset, model-based behavior is reflected as the probability of repeating the first-stage choice (stay) as a function of the occurrence of a transition from the previous trial (common, 70%; rare, 30%) and whether a reward was received (reward, nonreward). In a purely model-free learner, stay probabilities after reward should be higher than when no reward was presented regardless of transition type. In a purely model-based learner, stay probabilities after common-reward and rare-nonreward should be higher than common-nonreward and rare-reward. In our empirical data here, the stay probabilities obtained across conditions is a mix of both model-based and model-free behavior. Error bars reflect SEMs.

The sequence of events was presented in the same manner as a prior study (Eppinger et al., 2017), with the exception that we used the standard 70%/30% transition probabilities (whereas Eppinger et al., 2017 instead contrasted blocks of 60%/40% vs 80%/20%) and had a slightly shorted time to make a choice (1500 ms in this study vs 2000 ms in their article; Fig. 1). On each trial, participants were first presented with a fixation cross for 500 ms, and then shown a choice between two spaceships. They had 1500 ms to respond; after which, an outline over the chosen option would indicate their choice (feedback) for 500 ms. A fixation cross was shown for 500 ms before transition, where the transitioned planet was shown (a blank color block) for 1000 ms. Two aliens of that particular planet would then appear, with 1500 ms for choice, and with feedback of the chosen option subsequently shown for 500 ms. Each of the aliens led to a probabilistic reward with a picture of “space treasure,” or to no reward with a picture of “space dust,” that was presented for 1000 ms. Responses were indicated using the left (“Q”) and right (“P”) keys. The color of blocks behind rockets and those representing planets were randomized across all participants. Participants performed two blocks of 75 trials (i.e., 150 trials in total).

The task captures both model-based and model-free behavior. A participant who performs the task purely in a model-free way will make their first-stage choices solely on whether they were rewarded on the last trial (choosing the same option if rewarded previously), regardless of the transition type that occurred. In contrast, a model-based strategy will take into account both the history of reward and the transition structure of the task when making the first-stage choice. For instance, if a first-stage choice led to a rewarded second-stage option via a rare transition, a model-based learner would be more likely to choose the alternative first-stage choice on the next trial as a common transition would then lead to the previously rewarded second-stage option. However, a model-free learner would not make this adjustment in choice based on transition type, and instead would repeat the same first-stage choice again.

Before the experimental task, participants completed a tutorial that explained the key concepts of the paradigm: the probabilistic association between the aliens and rewards (10 trials) and the probabilistic transition structure of rockets to planets (10 trials). After this practice phase, they had to answer a three-item basic comprehension test regarding the key rules of the task. If participants failed to answer all questions correctly, the experimenter would reiterate the key concepts of the paradigm to the participant, allowing clarification.

##### Self-report psychiatric questionnaires, transdiagnostic dimensions, and IQ.

To quantify compulsivity in our sample, we applied a previously defined transdiagnostic definition (Gillan et al., 2016) that is based on a weighted combination of items drawn from nine self-report questionnaires (which were fully randomized). The questionnaires used were the Alcohol Use Disorder Identification Test (AUDIT), to assess alcohol addiction (Saunders et al., 1993); the Apathy Evaluation Scale (AES), to test for apathy (Marin et al., 1991); the Self-Rating Depression Scale (SDS), to test for depression (Zung, 1965); the Eating Attitudes Test (EAT-26), to test for eating disorders (Garner et al., 1982); the Barratt Impulsivity Scale (BIS-11), to test for impulsivity (Patton et al., 1995); the OCI-R, to test for OCD (Foa et al., 2002); the Short Scales for Measuring Schizotypy (SSMS), to test for schizotypy (Mason et al., 2005); the Liebowitz Social Anxiety Scale (LSAS), to test for social anxiety (Liebowitz, 1987); and the trait portion of the State-Trait Anxiety Inventory (STAI), to test for trait anxiety (Spielberger et al., 1983). The short IQ evaluation was the International Cognitive Ability Resource (I-CAR; Condon and Revelle, 2014). Questionnaires were fully randomized in their presentation. Correlations between questionnaire total scores ranged greatly (*r* = −0.08 to 0.79).

We used weights derived from a previous study (Gillan et al., 2016) to transform our scores as our sample size had too low a subject-to-variable ratio (*N* = 192) for *de novo* factor analysis, compared with the original study (*N* = 1413). Consistent with the original study from which the weights were derived (Gillan et al., 2016), item 13 on the SDS was mistakenly phrased “I am restless and can't sleep” rather than “I am restless and can't keep still”. Prior studies have demonstrated the stability of the factor structure in new data, with and without this error (Rouault et al., 2018; Seow and Gillan, 2020). Consistent with prior work, the resulting dimension scores were moderately intercorrelated (*r* = 0.33–0.42).

##### Behavioral data preprocessing.

Individual missed trials and trials with very fast (<150 ms) reaction times at the first-stage (indicating inattention or poor responding) were excluded from analyses. A total of 1082 trials (3.76%) were removed across participants [per participant mean = 5.64 (3.76%) trials].

##### Quantifying model-based planning.

The extent to which participants exhibited model-based (goal-directed) behavior was estimated from the stay/switch behavior of the first-stage choice (see *Two-step reinforcement learning task)* using mixed-effects models written in R, version 3.6.0 via RStudio version 1.2.1335 (http://cran.us.r-project.org; RRID:SCR_001905) with the *glmer()* function from the *lme4* package (RRID:SCR_015654), with Bound Optimization by Quadratic Approximation (bobyqa) with 1e5 functional evaluations. The basic model tested whether participants' choice behavior to Stay (i.e., repeat a choice they made on the last trial; stay, 1; switch, 0) was influenced by the Reward of the previous trial (rewarded, 1; unrewarded, −1), the Transition [common (70%), 1; rare (30%), −1] and their Interaction (Fig. 1). Within-subject factors (the intercept, main effects of reward, transition, and their interaction) were taken as random effects (i.e., allowed to vary across participants). In R syntax, the model was: Stay ∼ Reward * Transition + (Reward * Transition + 1 | Subject).

As a model-based strategy depends on the history of reward and the transition structure, the extent to which MB planning contributed to choice was indicated by the presence of a significant interaction effect between Reward and Transition. Split half-reliability, where the data were split into two subsets (even vs odd trials) and correlated and adjusted with the Spearman–Brown prediction formula, was estimated for model-based planning. To test whether the compulsive dimension was associated with model-based deficits, we included the total scores of all three dimensions (AD, anxious depression; CIT, compulsive behavior and intrusive thought; SW, social withdrawal) as a *z*-scored fixed effect predictors into the basic model described above. The extent to which compulsivity is related to deficits in model-based planning was indicated by the presence of a significant negative Reward * Transition * CIT interaction.

##### Sensitivity to task structure: reaction time (RT).

Recent work has shown that one effective way to index an individual's sensitivity to the structure of the task is via RTs (Shahar et al., 2019). In a similar fashion, we conducted a mixed-effect linear regression of transition type (Transition: common, −1; rare, 1) on second-stage RT (S2-RT). In the syntax of R with the *lmer()* function and *lmerTest* package for statistical tests (RRID:SCR_015656; as with for all subsequent mixed-effect models), the model was as follows: S2-RT ∼ Transition + (Transition + 1 | Subject). We asked whether compulsivity was associated with a reduction in RT sensitivity to the transition structure (RT-Trans) with an interaction of the total scores of the three dimensions (AD, CIT, SW) as *z*-scored fixed-effect predictors into the original model above, indicated by the presence of a significant negative Transition * CIT interaction. We report the standardized β-coefficients and SEs (applicable for all subsequent regression analyses).

##### EEG recording and preprocessing.

EEG was recorded continuously using an ActiveTwo system (BioSemi) from 128 scalp electrodes and digitized at 512 Hz. The data were processed offline using EEGLab (Delorme and Makeig, 2004; RRID:SCR_007292) version 14.1.2 in MATLAB R2018a (MathWorks; RRID:SCR_001622). Data were imported using A1 as a reference electrode, then downsampled to 250 Hz and bandpass filtered between 0.05 and 45 Hz. Bad channels were rejected with a criterion of 80% minimum channel correlation. All removed channels were interpolated, and the data were rereferenced to the average. To remove ocular and other non-EEG artifacts, independent component analysis was run on continuous data with runica, principal component analysis option on, and its components were rejected automatically with the Multiple Artifact Rejection Algorithm (Winkler et al., 2011), an EEGLab toolbox plug-in, at a conservative criterion of >90% artifact probability. For all EEG analyses, other nonspecific artifacts were removed after epoching using a criterion of any relevant electrode examined showing a voltage value exceeding ±100 µV. If participants had a rate of >70% of total epochs failing this criterion, their data were excluded from all analyses (*N* = 4; as reported in *Participant exclusion criteria*). The remaining participants had a mean of 147.46 epochs left (SD = 2.98).

##### Single-trial analyses with EEG signals.

All analyses described below (including time–frequency single-trial analyses) were conducted with mixed-effects models. For every single-trial analysis, we excluded single-trial EEG estimates that were ±5 SDs away from the mean of the group. A maximum of <0.79% (*n* = 215) of the total trials across all participants were excluded for any measure. The regression MB estimate (defined in *Quantifying model-based planning*) was used as the individual between-subjects model-based estimate in all EEG analyses.

##### Sensitivity to task structure: P300 and transition type.

The P300 has well established sensitivity to stimulus probability (Polich and Margala, 1997), and prior research in healthy humans hypothesized the P300 as a sensitivity marker of state transition knowledge on the two-step task, although the directions of the reported effects have varied (Eppinger et al., 2017; Sambrook et al., 2018; Shahnazian et al., 2019). Likewise, here we sought to investigate whether the P300 would be sensitive to individual subjects' sensitivity to transition structure and whether the effects were linked to model-based planning/compulsivity.

We first measured the P300 component at four parietal electrodes over the topography of the stimulus-locked peak [D16 (CP1), A3 (CPz), B2 (CP2), A4); see Fig. 3*A*]. Data were epoched from −500 to 1700 ms relative to the onset of the second-stage stimulus (aliens presented) and baselined corrected from −200 to 0 ms. Stimulus-locked single-trial P300 amplitudes were estimated as the mean of ±100 ms around the individual's averaged latency of their positive peak within a search window of 250–1000 ms after stimulus onset. To eliminate amplitude biases associated with RT differences. We subsequently aligned the epochs [measured at A4, A5, A19 (Pz), A32, the response-locked peak; see Fig. 3*B*] to the time of choice. The response-locked P300 amplitude was quantified as the mean amplitude −100 to 0 ms before response. We also measured the build-up rate of the response-locked signal as the slope of a straight line fitted to each single-trial waveform using the interval –400 to –200 ms. To investigate whether the P300 was sensitive to rare versus common transitions and whether this depended on model-based control/compulsivity, we regressed both stimulus- and response-locked P300 measures against transition type (Transition: rare, 1; common, 0) interacting with *z*-scored MB estimates or compulsivity (CIT, controlled for the other psychiatric dimensions AD and SW), taking Transition and the intercept as random effects. In R syntax, the models were EEG ∼ Transition * MB + (Transition + 1 | Subj) and EEG ∼ Transition * (CIT + AD + SW) + (Transition + 1| Subj), respectively.

##### Time–frequency analysis.

EEG data were epoched for both the first and second stages of the task for time–frequency analyses [alpha power (9–13 Hz) and theta power (4–8 Hz)] detailed in the subsequent sections, as follows: –1700 to 2200 ms stimulus locked at the first-stage (rockets) as well as –2000 to 3500 ms stimulus locked at the second stage (aliens). Time–frequency calculations were computed using custom-written MATLAB (MathWorks) routines. The EEG time series in each epoch was convolved with a set of complex Morlet wavelets, defined as a Gaussian-windowed complex sine wave: *e*^(-^*^i^*^2*time*^^f^^)^ *e*^(-time^2/2σ^2)^, where *i* is the complex operator; time is time; and *f* is frequency, which increased from 2 to 40 Hz in 40 logarithmically spaced steps. σ defines the cycle (or width) of each frequency band and was set to cycle/2π*f*, where cycle increased from 4 to 12 in 40 logarithmically spaced steps in accordance with each increase in frequency step. The variable number of cycles leverages the temporal precision at lower frequencies and increases frequency precision at higher frequencies. From the resulting complex signals of every epoch, we extracted estimates of power. Power is defined as the modulus of the resulting complex signal: Ζ(time) (power time series: ρ(time) = real[*z*(time)]^2^ + imag[*z*(time)]^2^).

The stimulus-locked first-stage epoch was baselined corrected to the average frequency power for each frequency band examined (i.e., alpha or theta) from −400 to −100 ms (corresponding to first-stage fixation), while for the stimulus-locked second-stage epoch it used −1400 to −1100 ms [corresponding to second-stage fixation, before presentation of the colored squares (i.e., planets)] as the baseline. The latter baseline window was chosen as the color of the planets was predictive of the aliens; as such, choice-relevant neural activity may potentially emerge in the interval between the onset of the planets and aliens. For single-trial estimates of frequency power, as baselining with division induces spurious power fluctuations because of trial-to-trial fluctuations, power at each individual trial was baseline-corrected with the linear subtraction method (Cohen, 2014) with its corresponding baseline activity: [power(time) – power(baseline)], at each frequency, at each channel. For visualization purposes in the figures presented, power was normalized by conversion to a decibel scale: (10*log10[power(time)/power(baseline)]).

##### Sensitivity to task structure: alpha power and transition type.

We were also interested in the idea that more sustained postplanning processes may be important for explaining model-based deficits in compulsive individuals. As such, we focused on the posterior alpha band (9–13 Hz), which, in addition to reflecting surprising outcomes (Fouragnan et al., 2017), is considered a general marker of mental activity and attention (Laufs et al., 2003; Klimesch, 2012) and is suppressed in conditions where increased mental effort is needed (Stipacek et al., 2003; Pesonen et al., 2007). Much like the P300, we hypothesized that in model-based planners alpha power would be greater following rare transitions. Potentially reflecting more than just an acute surprise, we predicted that alpha power would show a more sustained pattern of increased suppression on rare versus common trials, which, speculatively, are the sort that might be required to correctly update the (alternative) top stage choices following reward receipt on those rare trials. As a putatively core constituent of model-based planning, we hypothesized that the degree of this alpha sensitivity to transition type would be associated with individual differences in model-based choice. Moreover, if individuals high in compulsivity have an impoverished model of the task, we predicted they would show reduced alpha sensitivity to the transition types.

Alpha power was measured at five parietal-occipital electrodes [A18, A19 (Pz), A20, A21, A31; surrounding A20 electrode; see Fig. 8*A*] in an epoch centered on the onset of the second-stage stimuli (aliens) and baseline corrected with activity before the onset of the transition (planets; see Time–frequency analysis). Single-trial stimulus-locked alpha power estimates were measured as the mean power ± 250 ms around the average latency of the negative peak, specific for each individual, and were found within a search window of 0–1000 ms after stimulus (alien) onset. We additionally obtained alpha power estimates quantified across four 1000 ms rolling time bins by the mean amplitude within each time window. We labeled the time bins as they began from transition (planet presentation) to the stimuli (aliens presentation) from 0 to 1000 ms, followed by three windows spanning choice to reward from 1000 to 2000 ms, 2000 to 3000 ms, and 3000 to 4000 ms. The same approach of mixed-effect models with P300 and transition type was used to examine the influence of model-based estimates/compulsivity on alpha power representation of rare versus common transitions, except for where Transition was coded differently (rare, −1; common, 1) for ease of interpreting the direction of interaction effects.

##### Cognitive control: theta power during choice.

Mid-frontal theta power (4–8 Hz) is a well established EEG signature of exerting “cognitive control” over lower-level impulses (Sauseng et al., 2010; Cavanagh and Frank, 2014), including pavlovian biases (Cavanagh et al., 2013). We therefore considered theta power as a candidate signature associated with implementing model-based decisions and overriding more habitual model-free choices. If deficits in model-based planning in highly compulsive individuals arise because of a failure of implementation, theta power during choice would be negatively linked to compulsivity.

For theta power (4–8 Hz), power estimates were measured at four frontal midline electrodes [C21 (Fz), C22, C23 (FCz), A1 (Cz); see Fig. 8*B*] at the first-stage (see *Time–frequency analysis*). The mean power ± 250 ms around the individual's average latency of the positive peak found within a search window 0–500 ms after stimulus onset was taken for every epoch. Similar to preceding analyses, we tested whether single-trial theta power at the time of first-stage choice was associated with individual differences in MB choice, RT-Trans, or CIT (controlled for AD and SW). We did this by taking each of them as a *z*-scored predictor variables in their own linear regression model of trial-by-trial theta power using the following notation in R, which allows for a random intercept for each subject, as follows: S1-Theta ∼ predictor variable + (1 | Subject). We also conducted a *post hoc* analysis to test whether theta modulates participants' trial-by-trial RT (S2-RT) sensitivity to transition (Transition: common, 1; rare, −1) by testing a model of S2-RT ∼ Transition * S1-Theta + (Transition * S1-Theta + 1 | Subject).

##### Specificity with psychiatric questionnaire scores versus transdiagnostic dimensions.

Additionally, we examined the advantages of using a transdiagnostic definition of compulsivity as opposed to investigating single psychiatric questionnaires. We repeated the above time–frequency analyses (alpha and theta) with the individual total questionnaire scores (QuestionnaireScore, z-scored) replacing the three psychiatric dimensions (CIT, AD, and SW) in their respective regression models detailed above. Separate mixed-effects regression models were performed for each individual questionnaire as the correlation across questionnaire scores were extremely high in some cases (ranging from *r* = −0.09 to *r* = 0.79) as opposed to the transdiagnostic analysis where all three dimensions [that correlated moderately (*r* = 0.33–0.42)] were included in the same model.

##### Supplemental analyses.

Finally, to ensure the specificity of any observed effects to the task events outlined above, we also tested for an association between model-based planning and compulsivity with our candidate EEG signatures in reverse. That is, we tested whether model-based planning and compulsivity were linked to (1) alpha power at the first stage or (2) theta power sensitivity to transition type at the second stage. See Figure 8, *A* and *B*, for the respective analyses.

For all analyses, we report the standardized β-coefficients and SEs.

##### Data availability.

The code and data to reproduce the main figures are available at https://osf.io/mx9kf/.

## Results

### Compulsivity and model-based planning

**Figure 2.**
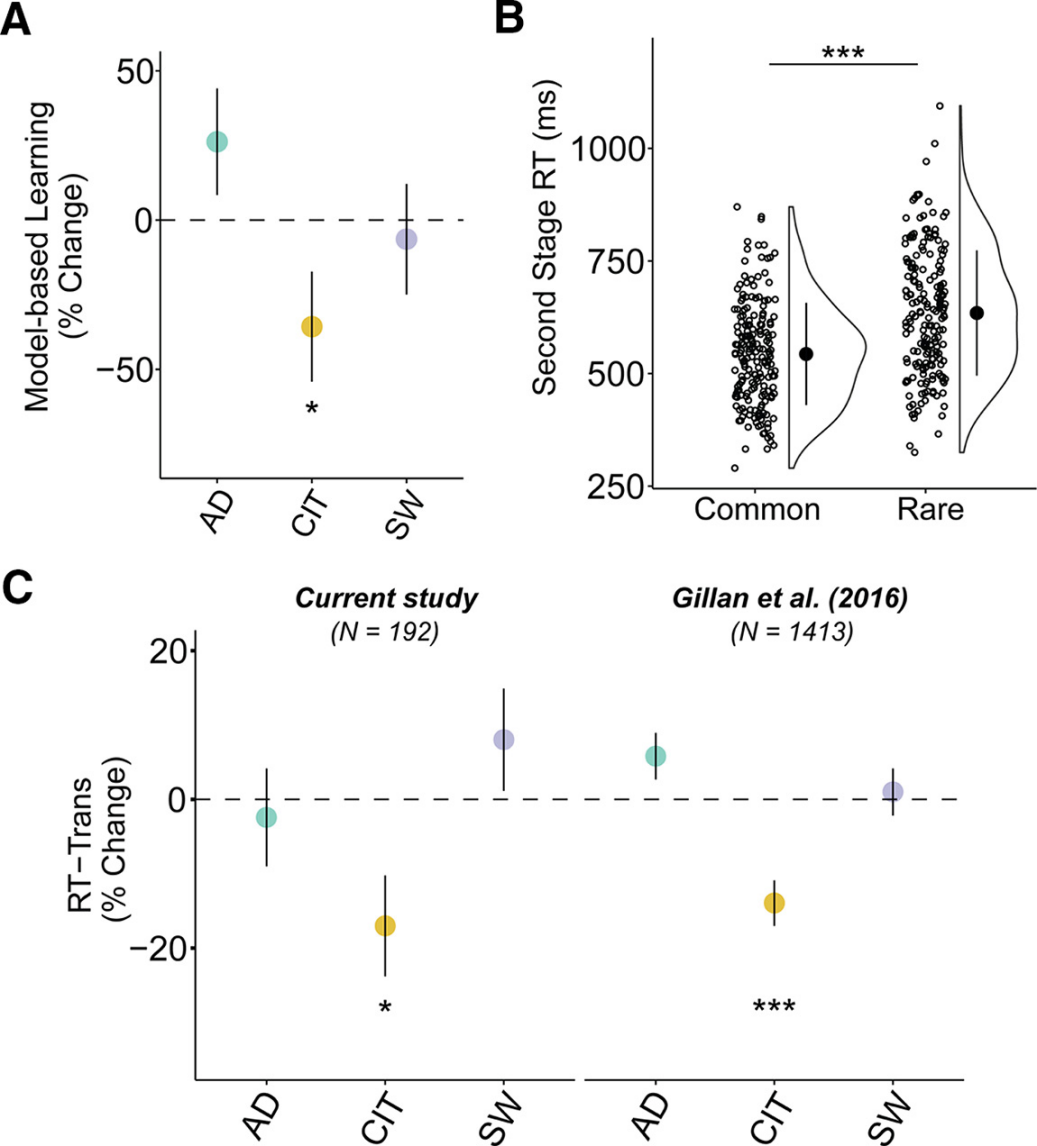
Model-based behavior and reaction times in compulsivity. ***A***, Model-based control estimated by a logistic regression of choice behavior with one-trial back reward and transition. Regressions were conducted in a model with all three dimensions: anxious-depression (AD), compulsive behavior and intrusive thought (CIT), and social withdrawal (SW). Model-based control is reduced in highly compulsive individuals. ***B***, Participants have on average a longer mean RT at second-stage choice after a rare transition than after a common one (paired *t* test: *t*_(191)_ = 16.16; 95% confidence interval, 79.85–102.05; *p* < 0.001). Circles in the raincloud plot (Allen et al., 2019) depict the mean RT of rare or common trials for each individual, with black marker indicating grand average mean and SD. ***C***, RT-Trans is diminished in highly compulsive individuals. We replicated the same effect in a prior dataset of *N* = 1413 (Gillan et al., 2016). For ***A*** and ***C***, error bars denote SE. The *y*-axes indicate the percentage change in model-based planning/RT-Trans as a function of 1 SD of psychiatric dimension scores. *p ≤ 0.05, ***p < 0.001.

Logistic regression analysis of choice behavior on the two-step task revealed clear evidence for model-based planning in this sample via a significant interaction between Reward and Transition (β = 0.20, SE = 0.03, *p* < 0.001; Fig. 1). Individual subject coefficients for this interaction term were extracted and used as an individual difference measure for EEG analysis (split half-reliability, *r* = 0.71). Consistent with prior work, there was also evidence for model-free learning, where subjects were more likely to repeat choices if they were followed by reward (main effect of Reward: β = 0.55, SE = 0.05, *p* < 0.001), and an overall tendency to repeat choices from one trial to the next (Intercept: β = 1.46, SE = 0.07, *p* < 0.001). Importantly, we replicated prior work in finding that individual differences in compulsivity and intrusive thought (hereafter called “compulsivity”) were associated with reduced model-based planning (β = −0.07, SE = 0.04, *p* = 0.05; Fig. 2*A*), while anxious depression (β = 0.05, SE = 0.04, *p* = 0.14) and social withdrawal were not (β = −0.01, SE = 0.04, *p* = 0.73).

### RT sensitivity to task structure

Someone who is aware of the task structure should expect to be presented with the second-stage state that is most commonly associated with their first-stage choice. As such, when a rare transition occurs, they will react to this violation in expectancy, requiring more time to respond and “replan” (Decker et al., 2016). We therefore hypothesized that participants would have a slower RT after a rare versus common transition and that this difference would be greater in participants who exhibit the most model-based behavior. We found support for both hypotheses; participants had a slower mean RT for rare versus common trials after transition (β = 0.17, SE = 0.01, *p* < 0.001; Fig. 2*B*) and this effect was larger in those with higher levels of model-based control (β = 0.28, SE = 0.07, *p* < 0.001). Crucially, we found that this effect was reduced in highly compulsive individuals (β = −0.03, SE = 0.01, *p* = 0.01; Fig. 2*C*). Prior studies using this task did not test for an association between compulsivity and this RT cost, but the data are readily available. To test the robustness of this finding, we therefore reanalyzed a prior dataset (*N* = 1413) collected entirely online (Gillan et al., 2016) using a similar variant of the two-step task and the same measure of compulsivity. We replicated this effect (β = −0.02, SE = 0.004, *p* < 0.001; Fig. 2*C*). This is, to our knowledge, the first evidence that compulsivity is associated with muted behavioral reactions to violations in transition expectancy, which is suggestive of disruption in the quality of the mental model of the task itself.

### P300 sensitivity to task structure

**Figure 3.**
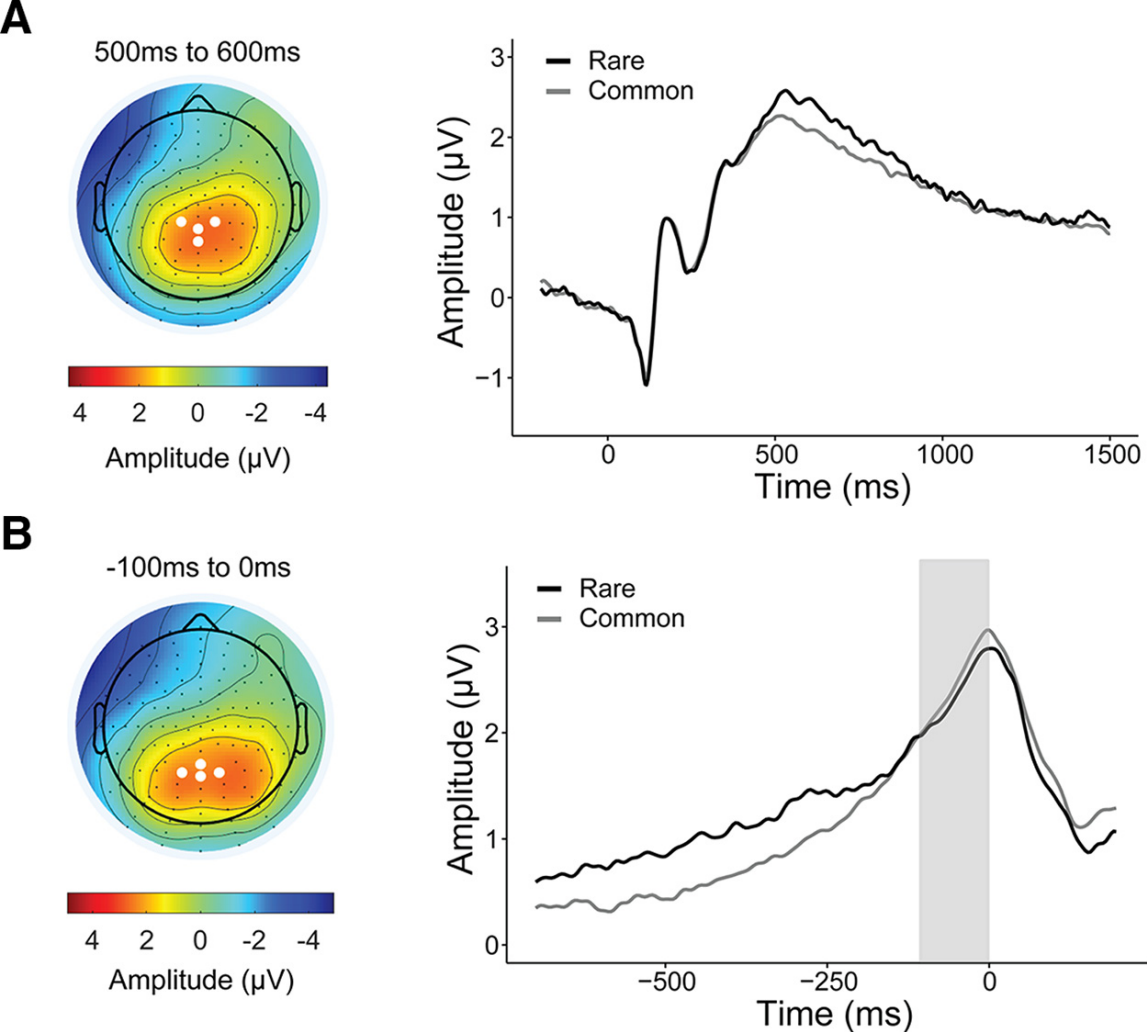
Second-stage P300 and transition type. ***A***, Grand average waveforms of rare and common trials stimulus locked to second-stage stimuli (aliens). Waveform is baselined −200 to 0 ms. The mean amplitude for stimulus-locked P300 was obtained over four centro-parietal electrodes [D16 (CP1), A3 (CPz), B2 (CP2), A4], as indicated by the white dots in the topography plot. This transition effect was no longer significant when the second-stage P300 signal was response locked (Fig. 3*B*). ***B***, Topography plot represents the P300 component –100 to 0 ms before second-stage response. White dots indicate parietal electrode sites [A4, A5, A19 (Pz), A32] where the positive component was measured. Grand average second-stage P300 is plotted response-locked comparing the waveforms following rare versus common transitions. Single-trial analyses indicate that the P300 amplitude, measured as the mean amplitude −100 to 0 ms (shaded gray), does not distinguish transition type (β = −0.02, SE = 0.01, *p* = 0.23).

The P300 or P3b has well established sensitivity to stimulus probability, exhibiting larger peak amplitudes for less probable stimuli (Polich and Margala, 1997). Prior research in healthy humans thus hypothesized that the P300 may be a marker of sensitivity to state transitions on the two-step task, though these studies have yielded inconsistent results, with some finding greater P300 amplitudes for rare versus common transitions (Sambrook et al., 2018; Shahnazian et al., 2019) and one finding the opposite (Eppinger et al., 2017). Here, we examined the second-stage stimulus-locked P300 and found a significant main effect of transition type (β = 0.03, SE = 0.01, *p* = 0.02), consistent with the studies by Sambrook et al. (2018) and Shahnazian et al. (2019) whereby greater P300 amplitude was observed after rare versus common transitions (Fig. 3*A*). However, this differential rare versus common signal was not larger in individuals high in model-based planning (β = 0.01, SE = 0.01, *p* = 0.35), nor did it show any association to compulsivity (β = 0.02, SE = 0.02, *p* = 0.24).

Recently, it has been suggested that P300 is more accurately characterized as a response-locked signal (O'Connell et al., 2012; Twomey et al., 2015). This raises the possibility that the stimulus-locked signal measurements favored in previous studies of the two-step task may have yielded cross-condition effects that were partly or entirely determined by RT differences. In light of these considerations, we complemented the stimulus-locked analyses with a response-locked version. When we repeated the analysis using response-locked P300 amplitude, we found that the transition effect was no longer significant and its direction was in fact reversed (β *=* −0.02, SE = 0.01, *p* = 0.23; Fig. 3*B*). Again, there was no association with model-based planning (β = −0.01, SE = 0.01, *p* = 0.49) or compulsivity (β = 0.01, SE = 0.02, *p* = 0.67). We also examined the build-up rate of the response-locked P300 as a measure of how quickly evidence for the decision was accumulated (Kelly and O'Connell, 2013). The build-up rate was steeper for common versus rare trials (β = −0.04, SE = 0.01, *p* = 0.002), but this measure was again not linked to model-based planning (β = −0.01, SE = 0.01, *p* = 0.46) or compulsivity (β = 0.01, SE = 0.02, *p* = 0.25). Thus, we concluded that the P300 may not provide the most reliable or sensitive measure of neural sensitivity to task structure.

### Alpha power sensitivity to task structure

Event-related potentials principally reflect activity changes that are short lived and strictly time-locked to particular events (Makeig and Onton, 2012). We investigated whether time–frequency measures such as alpha power (9–13 Hz), which has been previously linked to OCD (Perera et al., 2019), would allow us to capture a more sustained neural representation of the transition structure of the task. Specifically, we examined whether parietal-occipital alpha power locked to the second-stage stimulus was able to distinguish between rare and common transitions across a series of time bins in our task. This allowed us to ascertain not just whether participants showed sensitivity to task structure following a transition, but for how long they sustained that representation (e.g., as they made subsequent choices and received a reward). We reasoned that short-lived responses might reflect surprise stemming from arriving at a rare versus common state, but more sustained patterns could reflect postplanning processes required to update model-based top stage choice values.

**Figure 4.**
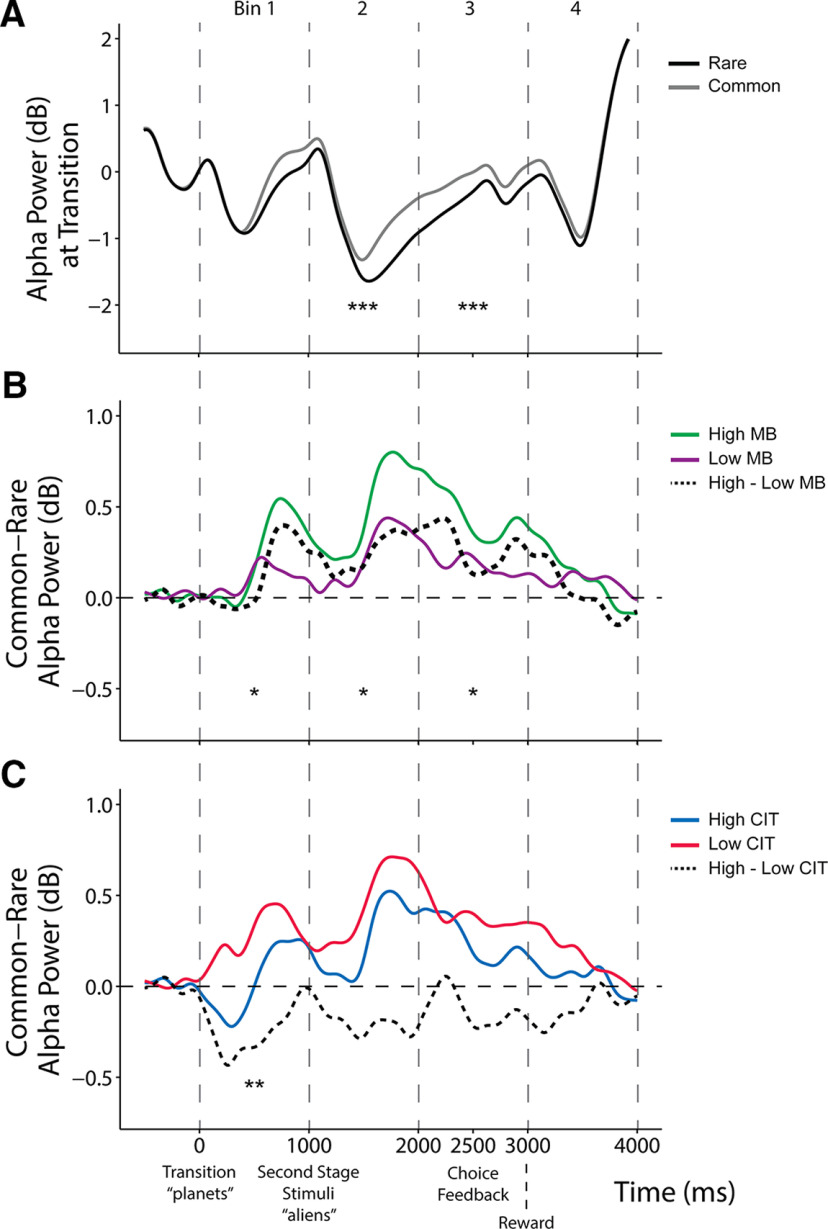
Stimulus-locked alpha power at transition. Alpha power was measured across four time bins of 1000 ms each separated by vertical dashed lines, starting from the transition (0 ms) until after reward (4000 ms), at parietal-occipital electrode sites (Fig. 8*A*). ***A***, Grand average second-stage alpha power waveforms between rare and common transitions. Continuous analyses revealed that the alpha difference (rare – common) is significant in time bins 2–3 (all β values > 0.03, SE < 0.01, *p* < 0.001). ***B***, Alpha power difference between transitions (common – rare) is depicted above by comparing top/bottom 50th percentile (*N* = 96/group) of participants grouped by MB estimates. Continuous analyses revealed that α difference (rare – common) is enhanced for more model-based participants in time bins 1–3 (all β values > 0.01, SE < 0.02, *p* < 0.05). ***C***, Alpha power difference between transitions (common minus rare) comparing top/bottom 50th percentile (*N* = 96/group) of participants grouped by or compulsivity (CIT). Continuous analyses revealed that αthe alpha difference (rare – common) is diminished for more compulsive participants in time bin 1 (β = –0.03, SE = 0.01, *p* = 0.007). Stars in time bins indicate significance from continuous analyses. *p < 0.05, **p < 0.01, **p < 0.001. These second-stage transition effects were specific to alpha power and were not present with theta power (Fig. 8*B*).

In line with our hypothesis, alpha power overall differentiated between the two transition types (β = 0.02, SE = 0.01, *p* < 0.001), such that parietal-occipital alpha was more suppressed after rare versus common transitions (Fig. 4*A*). We found that in a manner sustained over three rolling time bins beginning from the state transition (planet; 0–1000 ms: β = 0.02, SE = 0.01, *p* = 0.03) to the end of choice feedback (1000–2000 ms: β = 0.02, SE = 0.01, *p* = 0.03; 2000–3000 ms: β = 0.01, SE = 0.02, *p* < 0.05), individuals high in model-based control showed the largest alpha power differentiation (Fig. 4*B*). Importantly, this same signature was negatively related to compulsivity, with a significant association observed at the time after state transition (0–1000 ms: β = −0.03, SE = 0.01, *p* = 0.007; Fig. 4*C*). Overall second-stage alpha power was also associated with compulsivity (β = −0.09, SE = 0.03, *p* < 0.001); however, this effect was not related to model-based control (β = 0.03, SE = 0.02, *p* = 0.25) nor RT differences in transition types (β = −0.03, SE = 0.02, *p* = 0.20), highlighting that it is the sensitivity of alpha to task structure, not alpha overall, that best tracks model-based performance at this task.

**Figure 5.**
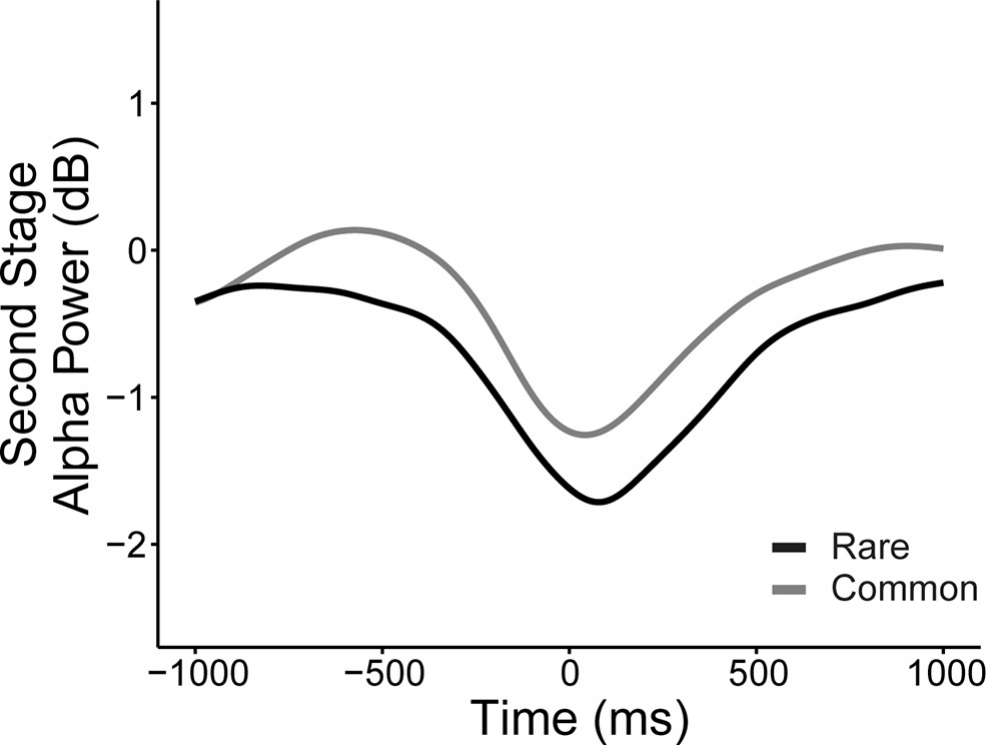
Grand average waveforms of rare versus common transitions for second-stage response-locked alpha power. RT differences between rare and common transitions were only significantly associated with stimulus-locked alpha power differentiation of states in the time bin before reward presentation (2000–3000 ms: β = 0.01, SE = 0.01, *p* = 0.04; all other time bins: *p* values > 0.30; Fig. 4). To complement our main result based on stimulus-locked alpha, we repeated the transition analysis with single-trial response-locked alpha estimates (measured as the mean of ±100 ms centered around each participant's averaged latency of the negative peak), which also yielded a significant association overall effect (β = 0.03, SE = 0.01, *p* < 0.001; Fig. 5). Similar to stimulus-locked alpha, rare transitions showed greater depression of alpha during choice selection for rare versus common transitions, suggesting that the alpha transition effect is not explained by RT.

**Figure 6.**
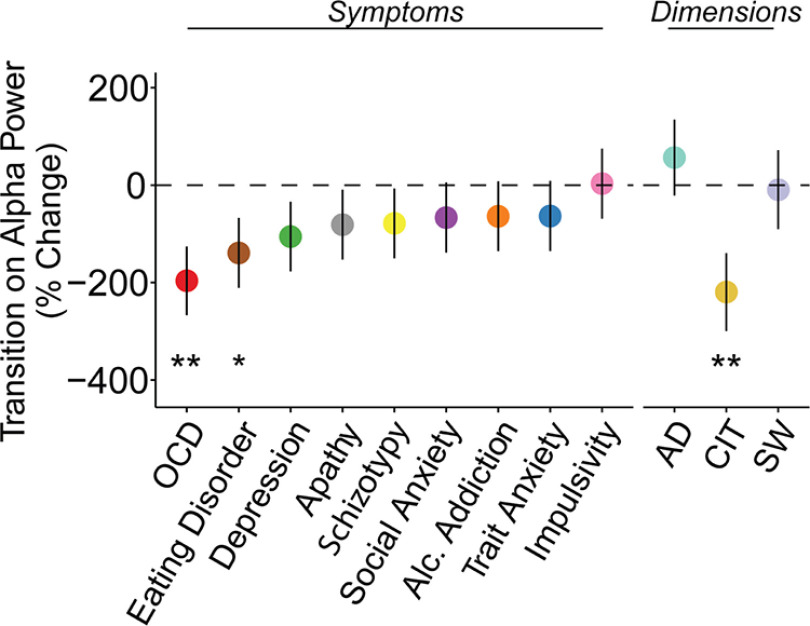
Second-stage alpha power sensitivity to transition at time bin 1 (0–1000 ms) with psychiatric symptoms and dimensions (anxious-depression (AD), compulsive behavior and intrusive thought (CIT) and social withdrawal (SW)). Alpha power differentiating rare versus common transitions was associated with both OCD and eating disorder symptoms. The transdiagnostic analysis showed the effect was captured by a compulsive dimension (CIT). The *y*-axis shows the percentage change in alpha power sensitivity to transition type (%) as a function of 1 SD increase of psychiatric questionnaire/dimension scores. Error bars denote SEs. *p ≤ 0.05, **p < 0.01.

Control analyses demonstrate that this transition sensitivity effect is present even if alpha estimates were locked to response times (Fig. 5) and is not found with second-stage theta power (which we examine later in the context of cognitive control at first-stage; see Fig. 8*B*). In terms of specificity to compulsivity, there were no associations to the other two transdiagnostic dimensions, anxious depression (β = 0.007, SE = 0.01, *p* = 0.47) or social withdrawal (β = −0.001, SE = 0.01, *p* = 0.91). When we examined the association between alpha band sensitivity to transition structure and all nine of the original psychiatric questionnaire total scores, we found diminished sensitivity in those with elevated OCD (β = −0.02, SE = 0.01, *p* = 0.006) and eating disorder symptoms (β = −0.02, SE = 0.01, *p* = 0.05; Fig. 6).

### Theta power at the time of choice

Finally, moving beyond participants' sensitivity to the transition structure of the task, we tested whether during the crucial time of first-stage choice, when model-based planning manifests in behavior, we could detect differences in a neural signature previously linked to cognitive control, mid-frontal theta (4–8 Hz). As theta has previously been shown to reflect computations crucial to goal-directed action (Sauseng et al., 2010; Cavanagh et al., 2013; Cavanagh and Frank, 2014), we hypothesized that model-based planning would be positively linked to theta power while compulsivity would be negatively associated with the neural oscillation.

**Figure 7.**
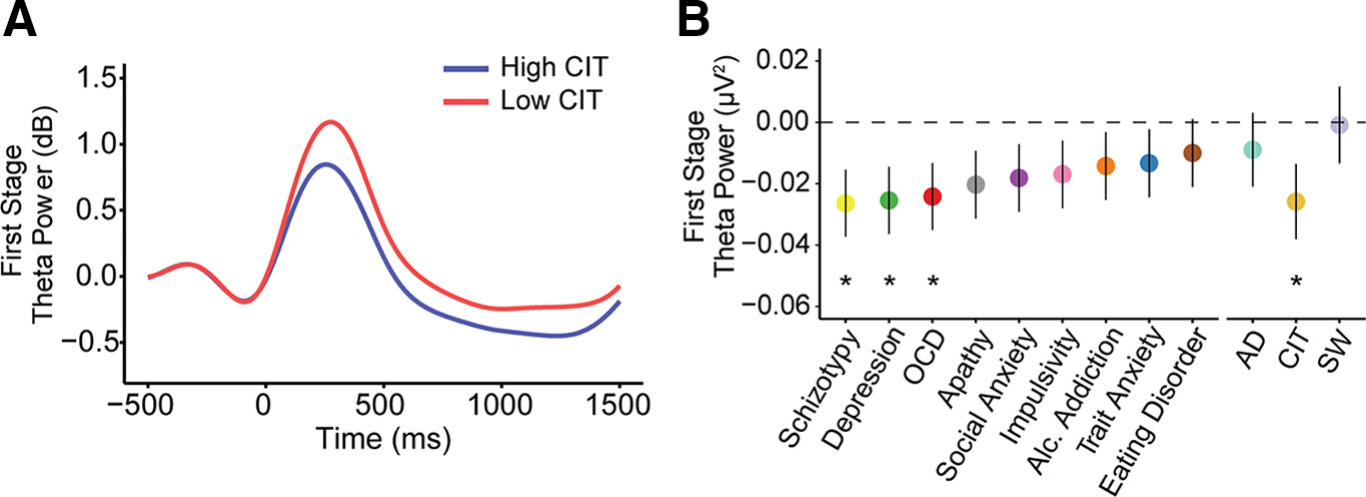
First-stage theta power with psychiatric symptoms and dimensions (anxious-depression (AD), compulsive behavior and intrusive thought (CIT) and social withdrawal (SW)). Theta power was measured at mid-frontal electrode sites (Fig. 8*B*). ***A***, Grand average waveforms of first-stage theta power comparing the top/bottom 50th percentile (*N* = 96/group) individuals based on their compulsivity (CIT) estimates. Single-trial analyses (with all participants) indicate that highly compulsive individuals exhibit a decrease in theta power (β = –0.03, SE = 0.01, *p* = 0.03). In contrast, first-stage alpha power was not associated with compulsivity (Fig. 8*A*). ***B***, Reduced theta power at first stage was linked to several questionnaire scores, but the effect was ultimately specific to compulsivity. The *y*-axis shows the change in theta power (in square microvolts) as a function of a 1 SD increase of psychiatric questionnaire/dimension scores. Error bars denote SEs. *p < 0.05.

We tested this using a mixed-effects regression analysis with trial-by-trial estimates of theta power as the dependent variable and individual differences in model-based choice (coefficients of the effect of reward * transition from the logistic regression of stay/switch behavior) as the predictor variable. Theta power during choice was not significantly associated with model-based planning (β = 0.02, SE = 0.01, *p* = 0.11), though, the trend was in the expected direction. When we used RT sensitivity to transition structure, instead of model-based choice, as an alternative manifest variable of the brain's capacity for model-based planning, we found a significant positive relationship with theta (β = 0.04, SE = 0.01, *p* = 0.002), indicating that those participants who had higher theta power during their first-stage choice also had larger differences in their RT between rare and common transitions at the second stage. Finally, using the same analysis approach, this time with individual differences in compulsivity as the predictor variable, we found an overall effect of lower theta at the time of choice in individuals high in compulsivity (β = −0.03, SE = 0.01, *p* = 0.04; Fig. 7*A*). Similar to alpha power modulations, reduced theta power at the first stage was linked to more than one questionnaire score—schizotypy (β = −0.03, SE = 0.01, *p* = 0.01), depression (β = −0.03, SE = 0.01, *p* = 0.02) and OCD (β = −0.03, SE = 0.01, *p* = 0.03)—and were associated with the compulsive dimension (β = −0.03, SE = 0.01, *p* = 0.03; Fig. 7*B*).

**Figure 8.**
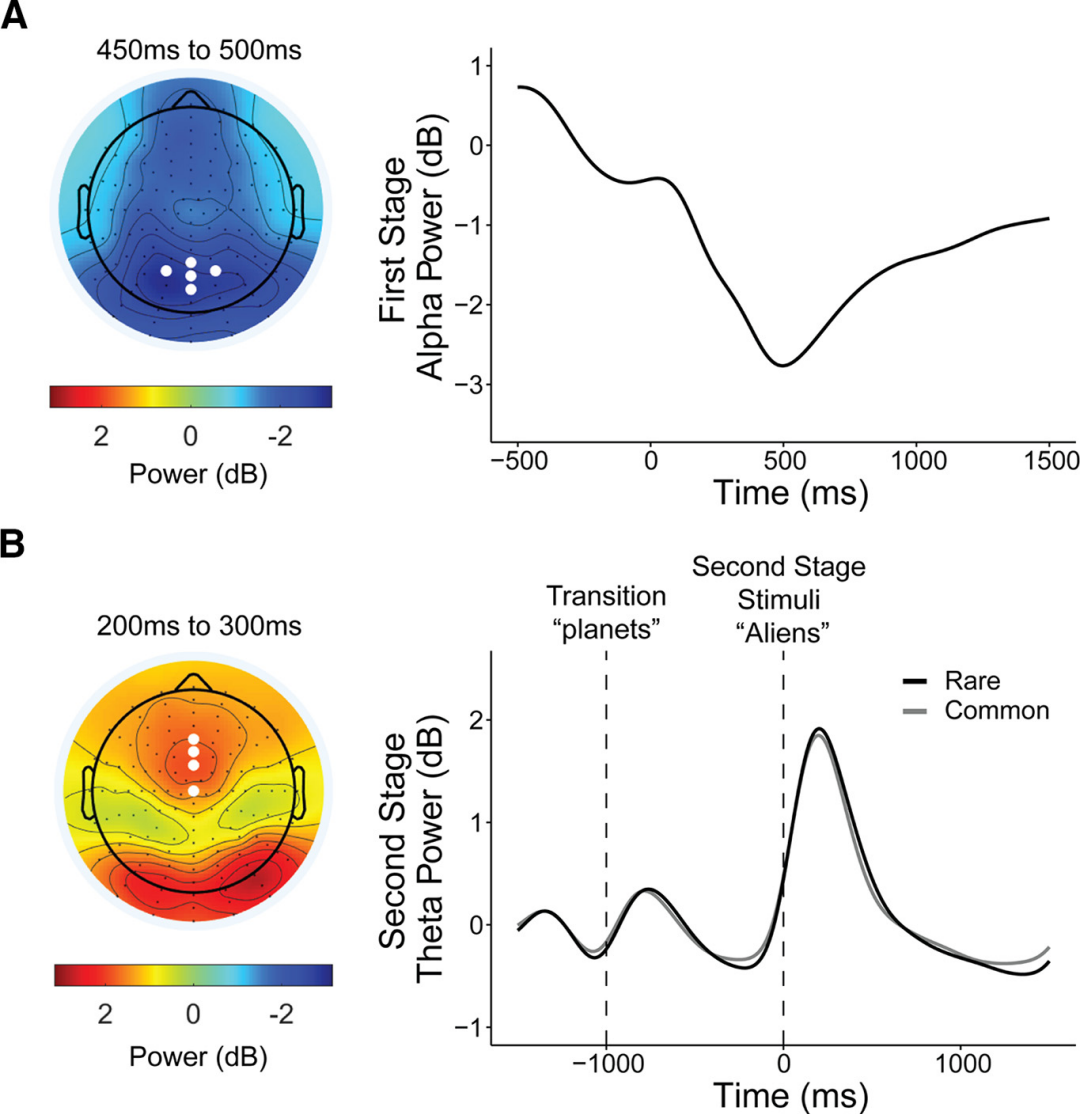
Supplemental analyses. ***A***, First-stage stimulus-locked alpha power. Topography and line plot (locked to first-stage rockets) show alpha depression during the making of a choice at the first stage. White dots on the topography plot indicate parietal-occipital electrode sites [A18, A19 (Pz), A20, A21, A31] where alpha was measured for both first and second stages. ***B***, Second-stage stimulus-locked theta power. Topography plot shows theta power increase after stimulus-onset at the mid-frontal scalp. White dots indicate electrode sites [C21 (Fz), C22, C23 (FCz), A1 (Cz)] where theta power was measured for both first and second stages. Theta power at the first-stage was not associated with compulsivity (β = −0.004, SE = 0.02, *p* = 0.84) or model-based planning (β = 0.01, SE = 0.02, *p* = 0.51). Theta power was also not linked to transition type (β = −0.01, SE = 0.01, *p* = 0.20) and had no transition interaction effects with compulsivity (β = 0.01, SE = 0.01, *p* = 0.14) or model-based planning (β = −0.004, SE = 0.01, *p* = 0.65).

One explanation for the somewhat closer association between theta and RT sensitivity (compared with model-based choice) is that theta at the time of choice might reflect participants' mental simulation of future states. We tested this *post hoc* using a within-subject analysis by examining whether on trials where theta was highest, subjects showed even greater RT sensitivity to transition type. We did not find evidence in support of this within-subject; the interaction between theta and transition type was not significant (β = 0.004, SE = 0.01, *p* = 0.57). Finally, by way of control analysis, we tested whether alpha power at first stage (Fig. 8*A*) was associated with compulsivity (β = −0.14, SE = 0.05, *p* = 0.002), model-based planning (β = 0.03, SE = 0.04, *p* = 0.45), or RT differences in transition types (β = −0.004, SE = 0.04, *p* = 0.92), but none were significant.

## Discussion

Model-based planning deficits linked to compulsivity have been theorized to arise from issues with the balance/arbitration between competing model-based and model-free influences during choice (Gillan and Robbins, 2014; Lee et al., 2014; Gruner et al., 2016; Lloyd and Dayan, 2019), but these presumed planning failures might, at least partially, arise from an impoverished internal model of task structure. Here, we found that highly compulsive individuals lacked neural and behavioral sensitivity to state transition probabilities, evidenced in their RT and parietal-occipital alpha power suppression in response to unexpected transitions. Speaking to the potential for more general cognitive control problems to also contribute to model-based deficits, we additionally took mid-frontal theta as its candidate neural signature and observed that highly compulsive individuals had reduced theta when they made their first-stage choices. These findings have important implications for refining theories of compulsivity, which may be associated with more fundamental problems in constructing and maintaining a model of the causal structure of the environment necessary for goal-directed “model-based” control than just cognitive control failures.

In line with prior research, participants exhibited longer RTs following rare transitions, which was also previously shown to relate to model-based planning (Deserno et al., 2015; Decker et al., 2016; Shahar et al., 2019). Crucially, the opposite was true of compulsivity, with the most compulsive individuals showing the smallest difference in RT between these trial types. This finding was robust—the effect replicates in a former dataset (*N* = 1413) tested online (Gillan et al., 2016). This may reflect a number of processes, including uncertainty arising from the presentation of unexpected options (Deserno et al., 2015) lower discriminability of the options presented following rare transitions (Shahar et al., 2019) or, as per our original hypothesis, a reduced awareness of the task structure (Decker et al., 2016) including action–state transitions necessary to build an accurate causal model of the world.

Moving beyond behavior, analysis of alpha power revealed a similar picture. Much like RT, alpha suppression at the second stage was sensitive to transition probabilities, with rare than common transitions associated with greater alpha suppression, possibly reflecting the greater mental effort required after rare transitions to call to mind action values associated with the unexpected options presented. In line with this, previous studies using *n*-back paradigms have shown greater parieto-occipital alpha suppression when working memory load increases (Stipacek et al., 2003; Pesonen et al., 2007). Importantly, this mental activity was sustained beyond second-stage choice right up until reward receipt, which might reflect that one must not only replan, but also that task structure information is used together with trial outcome to update first-stage choices. Consistent with this interpretation, individual difference analysis demonstrated that this difference in alpha suppression had important behavioral correlates. Model-based planners showed the largest differences in alpha power between transition types, while higher levels of compulsivity were associated with less of a distinction in alpha power between transition types. Building on the RT findings, we present neural evidence that compulsivity may be characterized by failures in representing the kind of causal action–state relations necessary to behave in a model-based manner. The notion that sustained alpha differentiation across common/rare trials reflects a postplanning process is speculative, and future research should aim to distinguish this from the effects of surprise.

Our data do not exclude the possibility that compulsive individuals also face issues with implementing model-based planning even when they have the requisite state knowledge. Indeed, we also found that mid-frontal theta, which is thought to support adaptive cognitive control in a variety of contexts (Cavanagh et al., 2012), was reduced in compulsive individuals during first-stage choice. In addition to being negatively related to compulsivity, theta power was also elevated in those whose RT was most sensitive to task structure and trended toward being elevated in model-based planners, supporting the view that theta activity at the time of choice at least in part reflects mental operations relevant to executing a model-based plan. However, disentangling the specific theta-driven processes is beyond the scope of our current experimental design. Theta power at choice time could reflect a host of executive processes such as selecting between competing options (including suppressing distracting stimuli; Nigbur et al., 2011), inhibiting unhelpful associations (Cavanagh et al., 2013), and the mental simulation/search of future states (Doll et al., 2015).

Previous EEG studies of the two-step task (Eppinger et al., 2017; Sambrook et al., 2018; Shahnazian et al., 2019) showed that the P300 was associated with state transitions. However, the inconsistent effect direction raises doubt as to how these differences should be interpreted. Recent literature conceptualizes the P300 as an evidence accumulation process that builds toward a peak at choice time (Twomey et al., 2015), and, as such, variances in RT will influence the latency of the stimulus-locked P300 amplitude peak (Kelly and O'Connell, 2015). Our results comparing stimulus-locked and response-locked analysis approaches suggest that it is the build-up rate of the P300 that is sensitive to transitions and that previously reported stimulus-locked amplitude modulations are attributable to RT differences. We also found that none of the analyzed P300 metrics were predictive of individual differences in model-based planning.

In this study, we used a transdiagnostic compulsive dimension that was previously shown to provide the best mapping to model-based deficits in an online general population sample (Gillan et al., 2016). We replicated this finding here and extend it to EEG correlates of behavior, where our alpha and theta modulations were relatively nonspecific with respect to total scores on the set of questionnaires we administered, compared with our *a priori* dimensional factor compulsivity. This research pipeline illustrates how mental health dimensions may be defined in large online samples and then used in smaller studies that can avail of the harder tools of neuroscience, like EEG (Gillan and Seow, 2020). While the applicability of these findings to diagnosed patients cannot be established here, recent work suggests that the core mechanisms we capture in general population samples are broadly equivalent, at least in compulsivity. For example, model-based deficits in diagnosed patients are also linked to individual differences in self-reported compulsivity irrespective of their specific diagnosis (e.g., whether they had an OCD diagnosis; Gillan et al., 2020). As such, there is growing evidence that the specific associations between cognition and compulsivity observed in the general population are likely clinically relevant.

Overall, our findings suggest that model-based difficulties in compulsivity may be linked to an impoverished mental model of environmental contingency—an interpretation bolstered by recent findings implicating diminished transition learning in compulsivity in a task devoid of value representations (Sharp et al., 2020). Future work should carry on in this vein, perhaps asking: are failures in memory encoding or retrieval responsible for model-based planning deficits in compulsivity? Are these effects specific to learning about actions and their consequences, or more distributed failures to learn about causality? Moreover, there are several facets of model-based planning beyond the learning/maintaining of the transition structure that may also be implicated, like the inhibition of opposing model-free signals at choice time, forward simulation of future states, attention to reward receipt and using that information for updating the action value options. Understanding these factors will provide a clearer picture of the neural mechanisms that lead to compulsive disorders and, hopefully, provide scope for intervening more effectively. The clear advantage of the use of EEG here is its temporal resolution, which was crucial in allowing us to capture the sustained differentiation of alpha power to transitions. With this, of course, comes with a lack of spatial precision. Future work combining fMRI and EEG might prove fruitful, particularly for dissecting potentially multiple processes at the time of first-stage choice. Finally, there is growing recognition that the dichotomization of two decision systems is oversimplified; model-based/model-free processes are partially synergistic, overlapping in certain situations and/or hierarchically organized (Cushman and Morris, 2015; Balleine and Dezfouli, 2019; da Silva and Hare, 2020). Future research must go beyond dichotomized frameworks to advance our mechanistic understanding of how deficits in building a model of the world translate to behavior irregularities such as compulsive habits.

Our findings may have implications for understanding how compulsive behaviors and obsessive beliefs develop in concert, in a more integrated fashion than previously considered. Clinical cognitive models of OCD have long presumed that compulsions are performed to reduce anxiety induced by obsessive beliefs (Salkovskis and McGuire, 2003; Fisher and Wells, 2005), in contrast to a more recent hypothesis suggesting that obsessions are *post hoc* rationalizations to explain the performance of compulsive behavior (Gillan and Sahakian, 2015). These data may suggest that the hard distinction between obsessions and compulsions might be less clear than these models propose. Failures in accurately representing the relationship between actions and their consequences may be a common source of both compulsive habitual behaviors in OCD and also faulty metacognitive beliefs that form the basis of obsessions. One might imagine that with a less stable world model representation, the more likely it is that a patient may develop faulty beliefs and rely on habitual representations.
